# Neurological Manifestations of Acquired Immunodeficiency Syndrome

**DOI:** 10.7759/cureus.10449

**Published:** 2020-09-14

**Authors:** Otto Jesus Hernandez Fustes, Carlos Arteaga Rodriguez

**Affiliations:** 1 Neurology, Complexo Hospital de Clínicas - Universidade Federal do Paraná (CHC- UFPR), Curitiba, BRA; 2 Neurology, Universidade Positivo, Curitiba, BRA

**Keywords:** acquired immunodeficiency syndrome, neurological manifestations, stroke, headache

## Abstract

Objectives

To determine the neurological manifestations in AIDS patients with a recent diagnosis.

Materials and methods

A descriptive, retrospective study was carried out on 82 patients, admitted to the University Hospital in Curitiba for a period of 30 months, with positive serology for HIV, using the enzyme-linked immunosorbent assay (ELISA) method for diagnosis and confirmation.

Results

Of the 82 patients studied, 28 (34.1%) presented neurological manifestations; 16 were male (57.14%) and 12 female (42.85%). The age ranged from 22 to 79 years, with a median age of 35 years. Neurological manifestations were: headache (19.29%), motor deficit (19.29%), cranial nerve disorders (14.03%), epileptic seizures (10.52%), dementia (8.77%), coma (5.26%), ataxia (5.26%), mental confusion (5.26%), and tremor (3.5%). Cerebrovascular and neuroinfectious diseases (including neurotoxoplasmosis, bacterial and cryptococcal meningitis, and cerebral tuberculosis) were the most frequent diagnosis with 10 cases each (33.33%). Of the 28 patients, 10 (35.7%) died and 18 (64.28%) were discharged.

Conclusion

Based on the results found, it appears that one-third of AIDS patients with up to one year of diagnosis (34.14%) have some type of neurological manifestation, highlighting the importance of performing specific anamnesis and neurological examination in these patients.

## Introduction

The first case of acquired immunodeficiency syndrome (AIDS) was detected in 1982 by the US Centers for Diseases Control (CDC) and was accepted by the World Health Organization in 1985 [1]. In the first cases of the disease, *Pneumocystis carini* pneumonia indicated severe immunodeficiency in these patients. Thus, it became clear the importance of detecting the causal agent of immunosuppression to better understand the disease [2,3].

The human immunodeficiency virus (HIV) is a retrovirus, with a genome that carries genes for the synthesis of three basic proteins and at least five other regulatory proteins. It has gag codes for antigenic proteins, pol codes for polymerase, and env codes for the outer protein envelope. Thus, the great variability in the presentation of HIV is due to its viral envelope. There are subtypes of HIV: HIV-1 and HIV-2. HIV-2 has been found in West African patients and has the same genetic organization as HIV-1. What differentiates them are the glycoproteins in the envelopes [3].

The virus invades the immune system and causes selective depletion of CD4+ T lymphocytes, favoring the appearance of opportunistic infections. Acute HIV infection is characterized by a condition similar to infectious mononucleosis (mononucleosis-like syndrome), with typical symptoms of fever, malaise, and headache. In AIDS, on the other hand, in addition to opportunistic lung infections and neoplasms such as Kaposi's sarcoma, neurological manifestations (NM) are common, which include opportunistic infections of the central nervous system (CNS), cerebrovascular disease (CVD), and neoplasms, such as primary cerebral lymphoma [3,4].

In 10 to 20% of AIDS patients, NM are their first clinical manifestation and in the course of the disease, between 30 to 40% of patients have some NM. Also, when the data are based on anatomopathological findings, these figures can reach up to 75 to 90% of the cases [5,6]. The most common opportunistic infections of the CNS are tuberculous and cryptococcal meningitis, considered among the main causes of death in these patients [3,4].

Due to its complexity, multisystemic clinical manifestations and the evolution of laboratory tests for the diagnosis of the HIV/AIDS complex, several clinical and laboratory diagnostic criteria have been developed since 1985. Currently the most used is the Adapted CDC: Existence of two reagent screening tests or one confirmatory for detection of anti-HIV antibodies + Evidence of immunodeficiency: diagnosis of at least one disease indicative of AIDS and/or T CD4+ lymphocyte count less than 350 cells/mm3. In 1993, the CDC expanded its definition of AIDS to include all HIV positive people with CD4+ T cell counts below 200 per microL of blood or 14% of the total lymphocytes [2,3].

With the development of laboratory studies and greater information to the population, clinical manifestations of AIDS including neurological ones have become recognized as well as the importance of immediate diagnosis and treatment for opportunists infections, crucial to changing quality of life.

NM or complications can occur in HIV/AIDS from four mechanisms: direct effects of HIV like toxic effects of viral proteins and chronic inflammation induced by the infection, opportunistic infections, immune reconstitution inflammatory syndrome, and adverse effects of antiretroviral therapy. Depending on the underlying etiology and the region of the brain affected, we see different neurological symptoms and signs [4,5].

The objective of this research is to determine the NM in patients diagnosed with AIDS and their clinical evolution in hospital.

## Materials and methods

An observational, retrospective and descriptive study was carried out on the medical records of patients admitted to a University Hospital in southern Brazil, over a period of 30 months, through the services of Infectious Diseases and Neurology, diagnosed with AIDS and notified to the Hospital Infection Control Center. The diagnosis of AIDS during hospitalization was based on clinical criteria, positive HIV serology and confirmation by the enzyme-linked immunosorbent assay (ELISA) method; they did not receive antiretroviral therapy (TARV). The treatment and CD4 measurement were performed in outpatient follow-up.

A protocol was prepared for a retrospective study of the medical records of these patients, which included: demographic data, symptoms, signs and neurological diseases. For the diagnosis of NM, clinical history and confirmation by imaging examination and/or study of cerebrospinal fluid were considered. Data are presented in percentages and arithmetic mean. This research was not submitted to the Research Ethics Committee because this was a retrospective, observational and descriptive study and non-interventionist.

## Results

**Figure 1:**
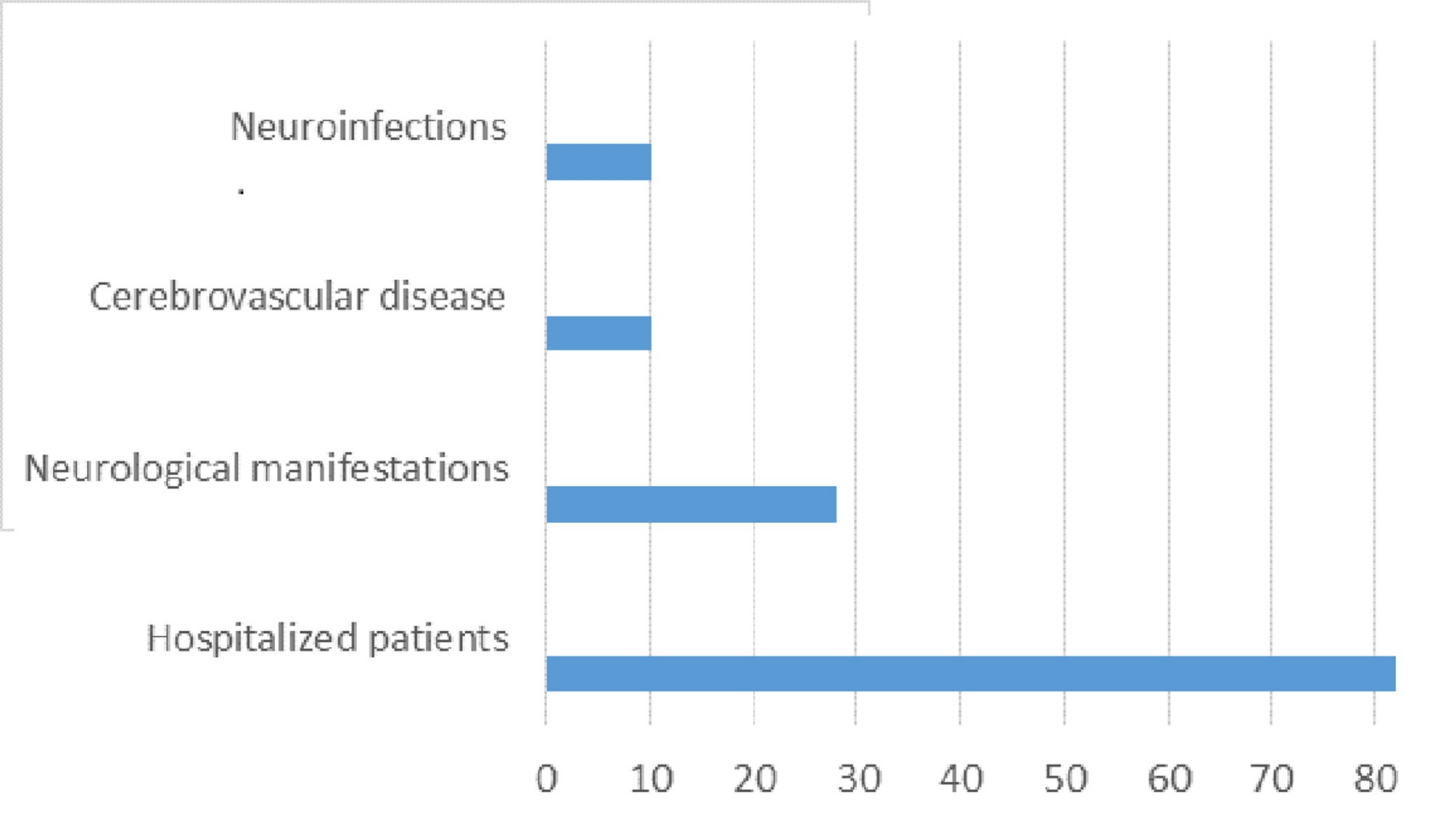
AIDS patients with neurological manifestations.

Of the 82 patients, 28 (34.1%) had neurological manifestations; of these 16 were male (57%) and 12 female (43%) (Figure 1). Patients with NM were between 22 and 79 years old (median 35 years old). The NM were: headache (21.42%), hemiplegia (21.42%), cranial nerve disorders (14.3%), epileptic seizures (10.71%), AIDS-dementia complex (7.14%), coma (7.14%), ataxia (7.14%), mental confusion (3.57%) and tremor (3.57%) (Table 1). Of the 28 patients with NM, 10 (37.8%) died.

**Table 1:** Neurological Manifestations

Neurological Manifestations	No. Patients	%
Headache	6	21.42
Hemiplegia	6	21.42
Cranial nerve disorders	4	14.28
Seizures	3	10.71
AIDS-dementia complex	2	7.14
Coma	2	7.14
Ataxia	2	7.14
Mental confusion	1	3.57
Tremor	1	3.57

Cerebrovascular disease (CVD) and infections of the central nervous system (neurotoxoplasmosis, bacterial meningitis, neurocryptococcosis, and neurotuberculosis) were diagnosed in 10 patients (35.7%) each. Of the 10 patients with CVD, six were men, aged between 22 and 63 years (mean of 37.82 years). CVD was ischemic in 90% and hemorrhagic in 10%, and 40% mortality was noted in the CVD subgroup.

## Discussion

It is known that HIV is neurotropic, neuroinvasive, and neurovirulent, which results in primary MN caused directly by the virus (invading microglia, astrocytes, macrophages, capillary endothelium) and secondary by immunodeficiency (opportunistic infections, brain tumors) and treatment-related. Thus, the interest, relevance, and complexity of MN in the course of HIV/AIDS is understood [5,6].

The numbers of NM in AIDS patients vary depending on the diagnostic criteria and stage of the disease. Nakagawa et al. 1997, in Japan, reported 28.7% of MN in AIDS patients [7]. Puccioni et al. in 1989 in Brazil, in a study similar to ours, found 15.71% of NM in young adults with less than a year of evolution of AIDS [8]. Another Brazilian study on inpatients reported 40% of NM in AIDS patients [9]. In our study, one-third of the cases studied (34.1%) had MN, without significant differentiation in relation to sex and with a predominance in younger patients (mean age 39 years).

The main NM found by our team were headache, motor deficit, cranial nerve palsy, epileptic seizures, AIDS-dementia complex, and changes in consciousness. Other researchers report the most frequent neurological symptoms and signs: motor deficit, headache, mental confusion, meningeal signs, and altered level of consciousness [8].

Fragoso et al. reviewed 50 hospitalized patients with AIDS and NM, reporting the incidence of headache 40% with or without other neurologic symptoms and signs. Although no large scale epidemiological study on the prevalence of headache in AIDS patients has ever been undertaken, it is generally accepted that HIV-infected individuals have a higher prevalence of headache than the general population [9].

CVD is a frequent cause of morbidity/mortality in AIDS patients and there are data in the literature that suggest an increase in CVD in AIDS patients, as compared to the general population. HIV causes cerebral arteriopathy characterized by occlusive and aneurysmal lesions [10,11,12]. In our series, 10 patients (33.3%) had CVD (six men) aged 22 to 63 years (mean of 37.82 years), ischemic in 90% and hemorrhagic in 10%, causing mortality in 40%.

Adamson et al. reported that the longer the survival of these patients, the greater their chances of developing neurological disease. In their study, 50% of patients had neurological manifestations, 68% of which were due to neurotoxoplasmosis [13]. Dannemann et al. also found *Toxoplasma gondii* encephalitis as the most frequent brain injury [14]. Simpson et al. agree with the authors above and adds that this entity can appear in 3 to 40% of the cases and it is usually due to a reactivation of a pre-acquired endogenous infection [4]. Neurotoxoplasmosis usually occurs in advanced cases of AIDS, when the CD4+ count is less than 200 cells/mm3. The most commonly reported manifestation is headache (55%), confusion (52%), fever (47%) and lethargy (43%). Focal neurological signs present in 69% of patients include hemiparesis, ataxias, and cranial nerve involvement [4]. Satishchandra et al. found 46.3% of the patients presenting symptoms resulting from cryptococcal meningitis [1].

It is estimated that 866 thousand people are living with HIV in Brazil and the epidemic in the country is considered stabilized, but there has been an increase in cases among young people. In 2017, 42,420 new HIV cases and 37,791 AIDS cases were diagnosed [15]. Our state of Paraná, southern Brazil, from January 2007 to December 2018, had an accumulation of 20,283 AIDS cases. The largest number is concentrated in the age group of 30 to 39 years, with 54.6% of cases. As for the number of people infected with the HIV virus, there were 18,763 records from 2007 to 2017 in the state [16].

The main focus of attention on this topic continues to that of early diagnosis and rapid access to treatment for patients with HIV, thus decreasing the risk of developing opportunistic infections and neurological complications.

## Conclusions

Some limitations of this study are the data are from a single teaching hospital in Southern Brazil. As a retrospective study, it is not possible to evaluate if preventive measures were implemented to the prevalent complications. It is extremely important to study in detail the manifestation of the disease in all organic systems, such as the nervous system. Thus, early diagnosis and specific therapy for these changes bring the patient a better quality of life.
